# Draft Genome Sequences of 10 Bacteria from the Marine *Pseudoalteromonas* Group

**DOI:** 10.1128/MRA.00404-21

**Published:** 2021-08-12

**Authors:** Amanda T. Alker, Bhumika S. Gode, Alpher E. Aspiras, Jeffrey E. Jones, Sama R. Michael, David Aguilar, Audrea D. Cain, Alec M. Candib, Julian M. Cizmic, Elise A. Clark, Alyssa C. Cozzo, Laura E. Figueroa, Peter A. Garcia, Casey M. Heaney, Alexandra T. Levy, Luke Macknight, Anna S. McCarthy, John P. McNamara, Kelvin A. Nguyen, Kendall N. Rollin, Gabriella Y. Salcedo, Julia A. Showalter, Andrew D. Sue, Tony R. Zamro, Tiffany L. Dunbar, Kyle E. Malter, Nicholas J. Shikuma

**Affiliations:** a Department of Biology, San Diego State University, San Diego, California, USA; b Viral Information Institute, San Diego State University, San Diego, California, USA; Loyola University Chicago

## Abstract

Here, we report the draft genome sequences of 10 marine *Pseudoalteromonas* bacteria that were isolated, assembled, and annotated by undergraduate students participating in a marine microbial genomics course. Genomic comparisons suggest that 7 of the 10 strains are novel isolates, providing a resource for future marine microbiology investigations.

## ANNOUNCEMENT

The genus *Pseudoalteromonas* comprises numerous marine species that are found in association with marine plants and animals (1). Some *Pseudoalteromonas* species produce compounds that inhibit the fouling of marine surfaces by invertebrates and algae (2), while others stimulate the metamorphosis of tubeworms, urchins, and corals (3). Many pseudoalteromonads possess the ability to produce diverse specialized metabolites (4–6), providing an understudied resource for biotechnology.

To engage undergraduates in discovery-based research, 10 purified isolates were cultured, and their genomes were sequenced, assembled, annotated, and analyzed by students participating in a marine microbial genomics (MMG) course at San Diego State University. The strains were collected from various marine organisms or objects using sterile cotton swabs (Table 1). A single colony of each strain was obtained on marine agar 2216 (BD Difco, Franklin Lakes, NJ, USA) and incubated at 25°C for 24 to 48 h. Colonies were transferred to marine broth 2216 and incubated for 24 to 48 h at 25°C before storage and DNA isolation. Genomic DNA was extracted using a Quick-DNA fungal/bacterial miniprep kit (Zymo Research, Irvine, CA, USA). 16S rRNA gene (27F-1492R) Sanger sequencing (Eton Biosciences, San Diego, CA, USA) classified all strains as being within the *Pseudoalteromonas* genus (>98% identity, >97% coverage). DNA was submitted to the Microbial Genome Sequencing Center (Pittsburgh, PA, USA) for library preparation (Illumina DNA prep kit; San Diego, CA, USA) and whole-genome sequencing (NextSeq 550; Illumina), producing 2 × 150-bp paired-end reads. Reads were trimmed using Trim Galore v0.6.1 (7), assembled using Unicycler v0.4.8 (8), integrated in PATRIC v3.6.9 (9), and annotated using the NCBI Prokaryotic Genome Annotation Pipeline (PGAP) v5.1 (10) with default parameters. General features of each genome are listed in Table 1.

**TABLE 1:** Genome features and metadata of 10 *Pseudoalteromonas* strains^*a*^^,^^*b*^

Strain	Closest strain, assembly accession no.	ANI (%)	Genome size (Mb)	*N*_50_ (bp)	GC (%)	No. of raw reads	No. of contigs	Coverage (×)	Colony pigment	No. of specialized metabolite gene clusters	*macB* E value	*macS* E value	*macT* E value	Isolation origin	Isolation location	SRA accession no.	Genome accession no.
MMG001	Pseudoalteromonas luteoviolacea H33-S, GCF_001625695.1	99.4	6.19	166,341	42.0	4,233,854	155	164	Purple	25	0	0	2E-109	Tubeworm	Quivera Basin, San Diego	SRR14127474	JAGJEM000000000
MMG002	Pseudoalteromonas luteoviolacea H33-S, GCF_001625695.1	99.4	6.19	166,863	41.9	3,534,634	151	136	Purple	25	0	0	2E-109	Tubeworm	Quivera Basin, San Diego	SRR14127473	JAGJEL000000000
MMG005	Pseudoalteromonas aurantia S3895, GCA_005887285.1	78.6	5.66	173,183	40.7	2,304,625	96	98	Orange	21	0	0	2E-59	Tubeworm	Quivera Basin, San Diego	SRR14127467	JAGJEK000000000
MMG006	Pseudoalteromonas sp. S3178, GCA_005886985.1	92.7	4.27	184,297	39.4	2,153,867	61	123	Brown	6	NS	NS	NS	Sediment	Silver Strand, San Diego	SRR14127466	JAGJEJ000000000
MMG007	Pseudoalteromonas sp. S3178, GCA_005886985.1	92.6	4.08	210,295	39.5	1,278,590	64	81	Brown	5	NS	NS	NS	Intertidal boulder	Bird Rock, San Diego	SRR14127465	JAGJEI000000000
MMG009	Pseudoalteromonas luteoviolacea NCIMB1944, GCF_001625565.1	91.9	6.51	469,158	42.2	4,505,820	75	173	Purple	16	0	0	7E-105	Aquarium filter	Aquarium, San Diego	SRR14127464	JAGJEH000000000
MMG010	Pseudoalteromonas sp. H103, GCF_001469205.1	77.2	3.57	204,150	38.2	1,477,062	39	107	Pink	2	NS	NS	NS	Sea snail	Sunset Cliffs, San Diego	SRR14127463	JAGJEG000000000
MMG012	Pseudoalteromonas sp. MSK9-3, GCA_003590335.1	79.1	5.44	56,381	40.7	1,307,359	261	62	Orange	20	0	4E-134	2E-59	Yellow sponge	Sunset Cliffs, San Diego	SRR14127462	JAGJEF000000000
MMG013	Pseudoalteromonas citrea S2233, GCA_005887445.1	78.6	5.84	153,003	40.8	1,538,911	145	68	Green	17	0	8E-135	2E-59	Yellow sponge	Sunset Cliffs, San Diego	SRR14127461	JAGJEE000000000
MMG019	Pseudoalteromonas luteoviolacea IPB1, GCF_001696455.1	98.9	5.94	309,281	42.7	3,759,179	75	170	Purple	17	0	0	3E-96	Coral	Water Factory, Curacao	SRR14127460	JAGJED000000000

a ANI values are calculated with respect to the closest strain. The MAC protein sequences MacB (WP_039609830.1), MacS (WP_039609824.1), and MacT (WP_039609826.1) were searched against the genomes using tBLASTn (20).

b NS, not significant.

To identify and compare the genomes of the newly sequenced strains with their nearest publicly available genomes, we used the Mash/MinHash search (9, 11) and calculated the average nucleotide identity (ANI) using EZBioCloud (12). Of the 10 genomes, 7 possessed ANI values that are below the 95% threshold that delineates species (13), suggesting that they are novel isolates (Table 1). When grown on marine agar 2216, all strains possessed pigmentation (Table 1) (14). When analyzed using antiSMASH v5.0 (15), the genomes were found to possess from 2 to 25 specialized metabolite biosynthesis gene clusters (Table 1). Strains MMG009 and MMG019 possess the brominated marine pyrroles/phenols (*bmp*) gene cluster (16, 17), which can produce a compound capable of stimulating the metamorphosis of coral larvae (18). Of the 10 genomes, 7 were found to possess *macB*, *macS*, and *macT* genes that compose phage tail-like contractile injection systems (Table 1), which promote tubeworm metamorphosis and other host-microbe interactions (19). These genome sequences provide a valuable resource for studying the ecology of *Pseudoalteromonas* bacteria and advancing natural product biotechnology.

### Data availability.

The genome sequencing and assembly projects have been deposited in DDBJ/EMBL/GenBank under BioProject number PRJNA716944. See Table 1 for the SRA and GenBank accession numbers.

## References

[1] Holmström C, Kjelleberg S. 1999. Marine *Pseudoalteromonas* species are associated with higher organisms and produce biologically active extracellular agents. FEMS Microbiol Ecol 30:285–293. doi:10.1111/j.1574-6941.1999.tb00656.x. 10568837

[2] Holmström C, Egan S, Franks A, McCloy S, Kjelleberg S. 2002. Antifouling activities expressed by marine surface associated *Pseudoalteromonas* species. FEMS Microbiol Ecol 41:47–58. doi:10.1111/j.1574-6941.2002.tb00965.x. 19709238

[3] Cavalcanti GS, Alker AT, Delherbe N, Malter KE, Shikuma NJ. 2020. The influence of bacteria on animal metamorphosis. Annu Rev Microbiol 74:137–158. doi:10.1146/annurev-micro-011320-012753. 32905754

[4] Gobet A, Barbeyron T, Matard-Mann M, Magdelenat G, Vallenet D, Duchaud E, Michel G. 2018. Evolutionary evidence of algal polysaccharide degradation acquisition by *Pseudoalteromonas carrageenovora* 9^T^ to adapt to macroalgal niches. Front Microbiol 9:2740. doi:10.3389/fmicb.2018.02740. 30524390 PMC6262041

[5] Chau R, Pearson LA, Cain J, Kalaitzis JA, Neilan BA. 2021. A *Pseudoalteromonas* clade with remarkable biosynthetic potential. Appl Environ Microbiol 87:1–16. doi:10.1128/AEM.02604-20. PMC8105009 33397702

[6] Bowman JP. 2007. Bioactive compound synthetic capacity and ecological significance of marine bacterial genus *Pseudoalteromonas*. Mar Drugs 5:220–241. doi:10.3390/md504220. 18463726 PMC2365693

[7] Krueger F. 2015. Trim Galore: a wrapper tool around Cutadapt and FastQC to consistently apply quality and adapter trimming to FastQ files. https://www.bioinformatics.babraham.ac.uk/projects/trim_galore/.

[8] Wick RR, Judd LM, Gorrie CL, Holt KE. 2017. Unicycler: resolving bacterial genome assemblies from short and long sequencing reads. PLoS Comput Biol 13:e1005595. doi:10.1371/journal.pcbi.1005595. 28594827 PMC5481147

[9] Davis JJ, Wattam AR, Aziz RK, Brettin T, Butler R, Butler RM, Chlenski P, Conrad N, Dickerman A, Dietrich EM, Gabbard JL, Gerdes S, Guard A, Kenyon RW, MacHi D, Mao C, Murphy-Olson D, Nguyen M, Nordberg EK, Olsen GJ, Olson RD, Overbeek JC, Overbeek R, Parrello B, Pusch GD, Shukla M, Thomas C, Vanoeffelen M, Vonstein V, Warren AS, Xia F, Xie D, Yoo H, Stevens R. 2020. The PATRIC Bioinformatics Resource Center: expanding data and analysis capabilities. Nucleic Acids Res 48:D606–D612. doi:10.1093/nar/gkz943. 31667520 PMC7145515

[10] Tatusova T, Dicuccio M, Badretdin A, Chetvernin V, Nawrocki EP, Zaslavsky L, Lomsadze A, Pruitt KD, Borodovsky M, Ostell J. 2016. NCBI Prokaryotic Genome Annotation Pipeline. Nucleic Acids Res 44:6614–6624. doi:10.1093/nar/gkw569. 27342282 PMC5001611

[11] Ondov BD, Treangen TJ, Melsted P, Mallonee AB, Bergman NH, Koren S, Phillippy AM. 2016. Mash: fast genome and metagenome distance estimation using MinHash. Genome Biol 17:132. doi:10.1186/s13059-016-0997-x. 27323842 PMC4915045

[12] Yoon SH, Ha SM, Kwon S, Lim J, Kim Y, Seo H, Chun J. 2017. Introducing EzBioCloud: a taxonomically united database of 16S rRNA gene sequences and whole-genome assemblies. Int J Syst Evol Microbiol 67:1613–1617. doi:10.1099/ijsem.0.001755. 28005526 PMC5563544

[13] Thompson CC, Chimetto L, Edwards RA, Swings J, Stackebrandt E, Thompson FL. 2013. Microbial genomic taxonomy. BMC Genomics 14:913. doi:10.1186/1471-2164-14-913. 24365132 PMC3879651

[14] Paulsen SS, Strube ML, Bech PK, Gram L, Sonnenschein EC. 2019. Marine chitinolytic *Pseudoalteromonas* represents an untapped reservoir of bioactive potential. mSystems 4:e00060-19. doi:10.1128/mSystems.00060-19. 31213521 PMC6581688

[15] Blin K, Shaw S, Steinke K, Villebro R, Ziemert N, Lee SY, Medema MH, Weber T. 2019. antiSMASH 5.0: updates to the secondary metabolite genome mining pipeline. Nucleic Acids Res 47:W81–W87. doi:10.1093/nar/gkz310. 31032519 PMC6602434

[16] Agarwal V, El Gamal AA, Yamanaka K, Poth D, Kersten RD, Schorn M, Allen EE, Moore BS. 2014. Biosynthesis of polybrominated aromatic organic compounds by marine bacteria. Nat Chem Biol 10:640–647. doi:10.1038/nchembio.1564. 24974229 PMC4104138

[17] El Gamal A, Agarwal V, Diethelm S, Rahman I, Schorn MA, Sneed JM, Louie GV, Whalen KE, Mincer TJ, Noel JP, Paul VJ, Moore BS. 2016. Biosynthesis of coral settlement cue tetrabromopyrrole in marine bacteria by a uniquely adapted brominase-thioesterase enzyme pair. Proc Natl Acad Sci USA 113:3797–3802. doi:10.1073/pnas.1519695113. 27001835 PMC4833250

[18] Tebben J, Tapiolas DM, Motti CA, Abrego D, Negri AP, Blackall LL, Steinberg PD, Harder T. 2011. Induction of larval metamorphosis of the coral *Acropora millepora* by tetrabromopyrrole isolated from a *Pseudoalteromonas* bacterium. PLoS One 6:e19082. doi:10.1371/journal.pone.0019082. 21559509 PMC3084748

[19] Shikuma NJ, Pilhofer M, Weiss GL, Hadfield MG, Jensen GJ, Newman DK. 2014. Marine tubeworm metamorphosis induced by arrays of bacterial phage tail-like structures. Science 343:529–533. doi:10.1126/science.1246794. 24407482 PMC4949041

[20] Camacho C, Coulouris G, Avagyan V, Ma N, Papadopoulos J, Bealer K, Madden TL. 2009. BLAST+: architecture and applications. BMC Bioinformatics 10:421–429. doi:10.1186/1471-2105-10-421. 20003500 PMC2803857

